# Spoon nails: still seen in today's world

**DOI:** 10.1002/ccr3.1404

**Published:** 2018-01-31

**Authors:** Bushra Moiz

**Affiliations:** ^1^ Section of Hematology, Pathology and Laboratory Medicine The Aga Khan University Stadium Road Karachi Pakistan

**Keywords:** Iron deficiency anemia, koilonychia, malnutrition

## Abstract

Koilonychia is a nail abnormality characterized by thin, brittle, and spoon‐shaped nails. It is frequently observed in chronic iron deficiency secondary to malnutrition, chronic blood loss, or malabsorption. It may also be idiopathic or related to occupation and rare systemic disorders. Presence of koilonychia should prompt investigations for iron deficiency.

A 22‐year‐old female presented with weakness, weight loss, and low appetite for few months. She had past history of tuberculous spondylitis and was successfully treated with anti‐tuberculous drugs. However, she experienced a severe backache since then and became dependent on narcotics for pain management. She was living alone after her mother's death and was suffering from depression. There was no history of any blood loss. She had received several courses of parenteral iron therapy in the past few years. Physical findings included pallor, glossitis and angular cheilitis. Her nails were brittle, ridged, and spoon‐shaped (koilonychia) (see panels A and B in Fig. 1). Her BMI was 17.58 kg/m^2^ indicating mild thinness while rest of the physical examination was unremarkable. Complete blood counts showed hemoglobin 8.1 g/L, hematocrit 26.4, MCV 62 fL, MCH 19 pg, white blood cells 8.8 × 10^9^/L, absolute neutrophil count 4.2 × 10^9^/L, and platelets 565 × 10^9^/L. Peripheral film showed hypochromic microcytic erythrocytes and pencil cells (panel C in Fig. 1). Subsequent tests showed serum ferritin of 2 ng/mL. Upper GI endoscopy was advised but refused by the patient. Overall findings were consistent with chronic iron deficiency anemia. Probable cause was poor nutrition as a consequence of psychiatric illness and drug abuse. She was started on oral iron therapy, however, because of poor compliance she was switched to parenteral iron therapy.

**Figure 1:**
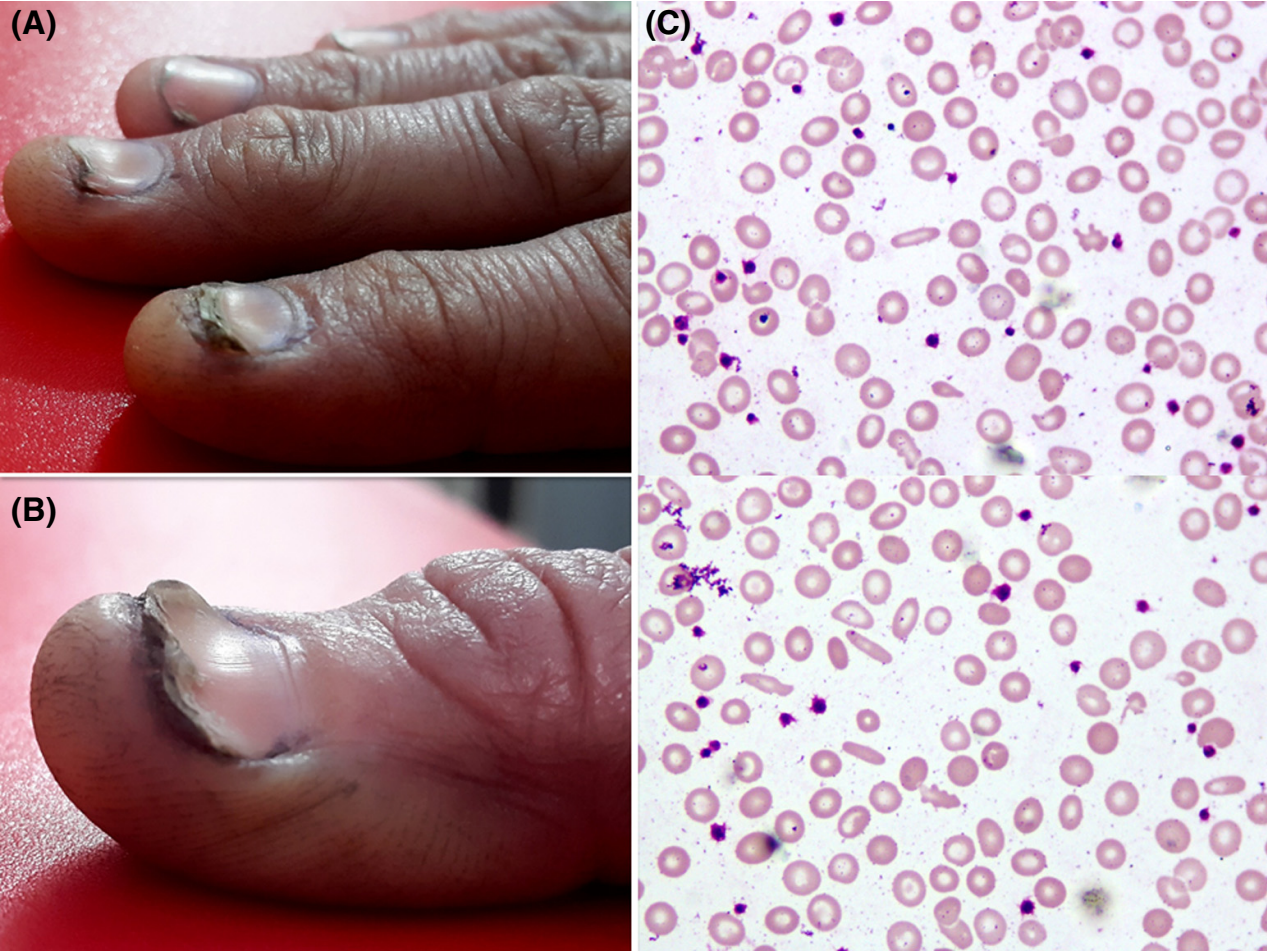
Panel A and B, respectively, shows koilonychia in fingers and thumb of right hand. Panel C shows peripheral smear demonstrating hypochromic microcytes with few tear drop cells and pencil cells typical of iron deficiency anemia.

Koilonychia is a physical finding of chronic iron deficiency anemia 1, 2. It results from malnutrition or chronic blood loss. Pathogenesis of koilonychia is not clear but may be related to reduced iron in iron‐containing enzymes in epithelial cells or poor blood flow with subsequent weakening and depression of underlying nail‐connective tissue 3. Our patient had poor nutrition and low compliance with iron therapy which resulted in koilonychia.

## Conflict of Interest

None declared.

## Authorship

BM: diagnosed the case, took photos and wrote the manuscript.
